# Antibiotic-Producing Beneficial Bacteria in the Gut of the Burying Beetle *Nicrophorus vespilloides*

**DOI:** 10.3389/fmicb.2019.01178

**Published:** 2019-05-31

**Authors:** Philipp Heise, Yang Liu, Thomas Degenkolb, Heiko Vogel, Till F. Schäberle, Andreas Vilcinskas

**Affiliations:** ^1^Department of Bioresources, Fraunhofer Institute for Molecular Biology and Applied Ecology, Giessen, Germany; ^2^Institute for Insect Biotechnology, Justus Liebig University Giessen, Giessen, Germany; ^3^Department of Entomology, Max Planck Institute for Chemical Ecology, Jena, Germany; ^4^German Center for Infection Research (DZIF), Partner Site Giessen-Marburg-Langen, Giessen, Germany

**Keywords:** symbiosis, gut microbiome, serrawettin W2, *Nicrophorus vespilloides*, burying beetle, nematodes, bioactivity-guided screening, natural products

## Abstract

The increasing prevalence of antibiotic-resistant human pathogens is a growing public concern and there is intense pressure to identify new antibacterial compounds that can be developed into antibiotics with novel mode of action. Evolutionary theory predicts that insects that have evolved to occupy sophisticated ecological niches by feeding and reproducing on carcasses will depend on their gut microbiome to prevent colonization by invading pathogens taken up with the diet. This inspired our hypothesis that the complex interactions between the core microbiome and the more flexible microbial communities dependent on the environment may promote the outsourcing of antibiotic synthesis to beneficial microbes. We tested this hypothesis by cultivating and characterizing bacteria isolated from the gut of the burying beetle *Nicrophorus vespilloides*, which feeds and reproduces on small vertebrate carcasses buried in the soil to avoid competitors such as fly maggots. The extracts of isolated bacteria were screened for activity against human pathogens such as *Escherichia coli*, *Pseudomonas aeruginosa*, *Staphylococcus aureus*, and *Candida albicans*. More than 400 strains were isolated, among which the crude extract of *Serratia marcescens* 2MH3-2 displayed promising activity against *Staphylococcus aureus*. Bioactivity-guided fractionation enabled purification of the primary antimicrobial compound of the extract. By LC-MS and NMR experiments, it was identified as serrawettin W2 (C_38_H_61_N_5_O_9_), the antibacterial and nematostatic activity of which was corroborated in our study. We postulate that this antibiotic could contribute to the control of both bacteria and phoretic nematodes in the gut, which compete for food when transferred to the carcass. Our study shows that the gut microbiome of *N. vespilloides* is a promising resource for the screening of antibiotic-producing bacteria.

## Introduction

The spread of antibiotic-resistant pathogens is a global challenge that causes up to 700,000 deaths per year, and mortality rates are expected to rise if novel antibiotics, particularly targeting Gram-negative bacteria, are not developed in the near future (Stern et al., 2017). New antimicrobials have been sought by screening industrial chemical libraries, but this has failed to provide new candidates. However, the vast majority of available antibiotics are natural products or derivatives thereof. Consequently, natural product research requires new strategies to revitalize declining natural product screening initiatives (Rossiter et al., 2017; Wright, 2017). One option is to expand the microbial strains used for natural product screening beyond *Actinomyces* and *Streptomyces* to include strains from microbial phyla that are neglected (Lasken and McLean, 2014). Another recent initiative focuses on the human gut microbiome as a resource for natural products (Wilson et al., 2017). This is based on the fact that a healthy gut microbiome can prevent invading pathogens colonizing the gastrointestinal tract, a phenomenon known as colonization resistance (Kim et al., 2017). Therefore, we explored the premise that particular insects, which are adapted to feed and reproduce on heavily-contaminated and microbe-rich sources, should harbor bacteria that produce antimicrobial compounds. Insects are particularly good candidates for discovering novel antibiotics and antibiotic producers due to their ancient mutualistic relationship with several microorganisms, which protect insect hosts against pathogens and parasites, thus allowing insects to colonize harsh environments, protect and preserve food resources, and prevent infection in their offspring (Flórez et al., 2017).

Burying beetles such as *Nicrophorus vespilloides* occupy a unique ecological niche by feeding and reproducing on carcasses, a nutrient-rich but ephemeral source that is subject to intense microbial competition (Vogel et al., 2017; Miller and Cotter, 2018). They display parental care by preparing the carcass as a diet for their offspring (Pukowski, 1933; Jacobs et al., 2016). This necessarily encompasses the regulation of the carcass-associated microbiome, which is used to transmit a core microbial community to their offspring (Shukla et al., 2018a; Wang and Rozen, 2018). Feeding and reproducing on carcasses requires adaptations to cope with both soil-borne and cadaver-associated microbes, which can harm also the eggs of *N. vespilloides* laid in close proximity to the decomposing carcass (Jacobs et al., 2014). *N. vespilloides* beetles are protected by a diverse spectrum of antimicrobial peptides, which are expressed in a context-dependent manner (Vogel et al., 2011; Jacobs et al., 2016) but they also sanitize the carcasses with oral and anal secretions consisting of at least 34 different small molecules with antimicrobial activity (Degenkolb et al., 2011). Four of these compounds were recently found to be produced by symbiotic *Yarrowia*-like yeasts that colonize the beetles’ hindgut (Vogel et al., 2017). This finding inspired our hypothesis that the *N. vespilloides* gut microbiome may also harbor beneficial bacteria producing low-molecular-weight bioactive compounds, as reported for other insect hosts (Kroiss et al., 2010; Flórez et al., 2017; Engl et al., 2018). However, competing organisms that live on the carcasses are not restricted to the kingdoms of bacteria and fungi, respectively (Shukla et al., 2018b), but also include phoretic nematodes that use burying beetles as a vehicle for transmission to carcasses used as a diet and for reproduction (Richter, 1993). A recent transcriptomic analysis of the carcass attended by *N. vespilloides* confirmed the transfer of living nematodes that are generally harmless but act as competitors for carcass-derived nutrients (Vogel et al., 2017). In contrast, other phoretic nematodes associated with this burying beetle (such as *Rhabditoides regina*) are parasites, with detrimental effects against the feeding larvae (Wang and Rozen, 2019).

To test our hypothesis that bacterial symbionts in the gut of *N. vespilloides* may provide protection against both competing microbes and phoretic nematodes, we isolated, cultured and screened bacteria from the beetle’s gut microbiome for the ability to inhibit (i) obligate and facultative human pathogens such as *Escherichia coli*, *Pseudomonas aeruginosa*, *Staphylococcus aureus*, *Candida albicans* and *Mycobacterium smegmatis*; and (ii) the model nematode *Caenorhabditis elegans*. We screened more than 400 isolates and culture extracts to identify symbiotic bacteria with the strongest activity against pathogens and nematodes, and used a combination of analytical techniques in an attempt to identify active compounds. Our findings allowed us to propose an ecological role for antimicrobial and nematicidal molecules in the novel context of *N. vespilloides* parental care.

## Materials and Methods

### Insect Rearing and Treatment

We used *N. vespilloides* beetles either caught in the wild during the summer in Giessen (Germany) or bred in captivity by H. Vogel (Max Planck Institute for Chemical Ecology, Jena, Germany). The wild beetles were caught by placing a dead mouse (*Mus musculus*) on soil in a modified container with a lid pierced to create a hole ∼3 cm in diameter. The container was buried with the lid at ground level and was checked daily. The captured beetles were first cooled to reduce movement and then washed sequentially in 1% H_2_O_2_, sterile water, 70% ethanol and again in water for 1 min each to reduce surface contamination risks. The surface-sterilized beetles were dissected and the entire gut (from foregut to rectum) was removed. Gut sections were macerated in sterile phosphate-buffered saline (PBS) using a sterile pipette tip, and each preparation was serially diluted. Fifty μl of the dilutions 10^-3^ to 10^-6^ were plated on brain heart infusion (BHI) medium (Carl Roth, Karlsruhe, Germany), water agar and Trypticase soy yeast extract (TSBYE; Sigma-Aldrich, Munich, Germany). Each medium was prepared with 1.5% agar. Additionally, the same media have also been used in parallel, supplemented with ampicillin and kanamycin (see next paragraph).

### Bacterial Cultivation and Identification

Microbes were cultivated on BHI agar or TSBYE agar for 1–4 days or on water agar for up to 3 weeks. Additionally, antibiotic-resistant cultures were selected in parallel on media supplemented with 0.05 mg/ml ampicillin and 0.025 mg/ml kanamycin. All cultures were incubated at 28°C. If growth was too rapid for colony selection, we reduced the temperature to 21°C (room temperature) and/or reduced the nutrient yield to 10% (e.g., for the genus *Proteus*). Colonies were visually selected, picked and streaked onto fresh agar until no contamination was detected by eye or microscopy. Isolates were then kept in Roti-Store cryovials (Carl Roth) at –80°C.

Isolates from the *N. vespilloides* gut were identified by 16*S* rDNA gene amplification using a C1000 Thermal Cycler (Bio-Rad, Hercules, CA, United States) followed by Sanger sequencing (Eurofins, Ebersberg, Germany). We used the standard primer set 27F (5′-AGA GTT TGA TCM TGG CTC AG-3′) and 1492R (5′-ACC TTG TTA CGA CTT-3′) (Lane, 1991). Each amplification was based either on a standard colony PCR or was achieved by first lysing the cells in 0.2% SDS. For the standard colony PCR, cell material was picked using a pipette tip and mixed with 30 μl double-distilled water before heating the cell suspension to 95°C for 5 min. We used 1–3 μl of each cell lysate as a PCR template.

The PCR comprised 32 cycles of denaturation (95°C, 5 min), annealing (56°C, 30 s) and extension (72°C, 1 min) followed by a final elongation at 72°C for a further 1 min before storage at 4°C. The PCR products were separated by 1.5% agarose gel electrophoresis at 110 V for 40 min, and product bands were isolated using a Gene Jet gel extraction kit (Qiagen, Hilden, Germany). After Sanger sequencing as above, the sequences were identified using the NCBI Basic Local Alignment Search Tool (BLAST).

MEGA 7 (molecular evolutionary genetics analysis) was used to align the obtained 16*S* gene sequences of the *Serratia* isolates to 16*S* sequences of *Serratia* type strains from the NCBI database. From this alignment, a maximum-likelihood phylogenetic tree was generated and circularized in order to improve its visibility.

### Chemical Extraction and Bioassay

We established three 100-ml cultures in 300-ml Erlenmeyer flasks from selected isolates based on 16*S* gene sequences. Fermentation of approximately 130 bacterial isolates was carried out at 28°C, shaking at 140 rpm for 9 days with three harvesting points (days 1, 6, and 9). The cells were harvested directly into liquid nitrogen and lyophilized after shell freezing. Extracts were prepared in 50 ml ethyl acetate with 15 min ultrasonication to break up the cells. After filtration, a second extract was prepared as described above, using methanol instead of ethyl acetate. Both crude extracts were then concentrated 100-fold and stored at 4°C.

The bioactivity tests were set up against clinically relevant strains and tested in duplicate using 10, 1, 0.5, and 0.25 μl of each crude extract in 96-well plates to ensure a high throughput. For *Staphylococcus aureus*, *E. coli*, and *C. albicans*, 100 μl of cation-adjusted Müller-Hinton broth was inoculated with ∼5 × 10^5^ cells/ml and incubated at 37°C for 18 h, shaking at 180 rpm. Growth inhibition was measured against both positive and negative controls at 600 nm (gentamicin serial dilution/fluconazole) using a plate reader.

*M. smegmatis* was grown for 48 h at 37°C in BHI medium supplemented with 1% Tween-80. Afterward, the cell suspension was diluted to ∼1 × 10^5^ cells/ml in cation-adjusted Müller-Hinton broth, and 100 μl of the diluted suspension was dispensed into white 96-well flat-bottom plates. The plates were incubated for 48 h at 37°C, shaking at 180 rpm. Cell viability was determined using the BacTiter-Glo^TM^ assay (Promega, Mannheim, Germany) and a LUMIstar OPTIMA microplate luminometer (BMG Labtech, Ortenberg, Germany) for the readout (Dardic et al., 2017).

### Antimicrobial Screening

The following strains were used to determine antimicrobial activity: *E. coli* ATCC 25922, *P. aeruginosa* ATCC 27853, methicillin-sensitive *Staphylococcus aureus* ATCC 25923 (MSSA), *C. albicans* (in-house strain) and *M. smegmatis* ATCC 607. The minimal inhibitory concentration (MIC) was also evaluated against *Bacillus subtilis* DSM 10, methicillin-resistant *Staphylococcus aureus* ATCC 33592 (MRSA) and *Listeria monocytogenes* DSM 20600.

### Fraction Collection and Analysis

Crude extracts were fractionated by high-performance liquid chromatography (HPLC) using a Dionex ICS 3000 instrument equipped with a Dionex Acclaim 120 C8 3 μm 2.1 × 150 mm column (Thermo Fisher Scientific, Waltham, MA, United States). Eluent A consisted of 1% formic acid in water and eluent B consisted of 1% formic acid in acetonitrile, flowing at a rate of 250 μl/min at a column temperature of 35°C. The elution profile was 20% B isocratic for 5 min, then a gradient from 20 to 100% B in 55 min, then 100% B isocratic for 15 min, and then a return to the initial conditions in 1 min followed by re-equilibration for 14 min. Fractions were collected during the first 75 min. We injected 10 μl of bioactive, filtered crude extract into the system for separation. Subsequently, the fractions were analyzed by high-resolution electrospray ionization quadrupole time-of-flight mass spectrometry (QqTOF-ESI-HRMS) using a Bruker Daltonics instrument (Bremen, Germany) running oTOF Control v3.4 and Compass v1.7. The instrument was equipped with an orthogonal ESI source. Source parameters were adjusted as follows: capillary voltage 4.5 kV, end plate offset 500 V, nebulizer gas 1.6 bar, dry gas 8 l/min with a dry temperature of 200°C. Samples were screened in positive-ion mode. The mass spectrometer was coupled to a Dionex UltiMate 3000 HPLC system running under Chromeleon Express (Thermo Fisher Scientific). Both instruments were controlled using HyStar v3.2 SR 4.

### FLASH Chromatography

Compounds were isolated following large-scale fermentation (5 l volume). The crude methanol extract was treated multiple times with acetonitrile to precipitate any proteins. The precipitate was filtered and dissolved in water, then freeze-dried and dissolved in methanol at a concentration of 50 mg/ml. The organic and aqueous phases (50 mg/ml) were again tested for bioactivity against *Staphylococcus aureus*. The bioactive extract was then mixed with an equal amount of Celite before drying in a rotary evaporator under reduced pressure. The Celite-sample mixture was used to prepare a pre-column. The samples were separated on a C18 reversed-phase column (Interchim PuriFlash C18-AQ 30 μM F0120) using water as eluent A and acetonitrile as eluent B. The elution profile was 20% B isocratic for 8 min, then a gradient from 20 to 100% B in 45 min, then 100% B isocratic for 10 min. Fractions were collected in a peak-dependent manner (i) at 210 nm, (ii) at 254 nm, and (iii) targeting peaks visualized using an evaporative light-scattering detector. The reaction tubes were combined in a logical order to reunite separated peak fractions and the solvents were evaporated. The remaining substances were then dissolved in methanol at 25 mg/ml and retested for antimicrobial activity using the growth inhibition assay described above.

### NMR Spectroscopy

The purified antimicrobial compound was analyzed by ^1^H and ^13^C NMR spectroscopy, as well as correlation spectroscopy (COSY), heteronuclear single quantum coherence (HSQC) spectroscopy and heteronuclear multiple-bond correlation (HMBC) spectroscopy using a Bruker AV400 device. Chemical shifts were referenced to the methanol-d6 solvent residual peaks, *δH* = 3310 ppm for ^1^H and *δH* = 49,000 ppm for ^13^C.

### Nematode Motility Assay

*Caenorhabditis elegans* was grown on nematode growth medium (NGM) covered with a lawn of *E. coli* OP20 for 4 days at 20°C. The worms were washed off the Petri dish into a 15-ml Falcon tube using a glass Pasteur pipette and M9 buffer. The nematode suspension was then centrifuged at 440 *g* for 2 min and the supernatant was removed. The pellet was washed again with M9 buffer and, after another step of centrifugation as described above, the content of the tube was reduced to 3.5 ml. To eliminate the worms and synchronize the suspension, we added 1.5 ml of bleach mix (0.5 ml 5 M NaOH, 0.5 ml NaOCl, and 0.5 ml water). The suspension was briefly mixed and shaken until the color of the solution changed from yellowish to clear (4–6 min). The tube was filled up to 15 ml with M9 buffer and centrifuged for another 4 min at 2760 *g*. The supernatant was quickly removed without touching the pellet until only 0.1 ml remained, and 15 ml of M9 buffer was added. In order to completely remove the bleach mix, the tube was carefully inverted and the pellet was rinsed three times with M9 buffer before centrifugation at 2760 *g* to remove all liquid. Finally, we added 10 ml of M9 buffer and 10 μl of cholesterol (5 mg/ml in 99% ethanol). Nematodes hatched overnight while the culture was shaking at room temperature. The nematode-containing medium was centrifuged for 4 min at 1200 *g* to remove all liquid. After a final washing step with 15 ml M9 buffer, the freshly hatched nematodes were centrifuged and the concentration was adjusted to 10 L1 nematodes per 100 μl by diluting with NGM seeding medium. The latter was prepared by supplementing 10 ml NGM with 10 μl 5 mg/ml cholesterol, 10 μl 25 mg/ml carbenicillin and 50 μl of an *E. coli* OP50 overnight culture.

The tests were carried out in triplicate in a 96-well plate with ivermectin (10 μg/ml in DMSO) as the positive control. Serrawettin W2 was readily isolated from a culture of *Serratia marcescens* 2MH3-2 and purified in the course of a bioactivity-guided screening as described. A stock solution in DMSO was serially diluted from 256 to 2 μg/ml. DMSO was used as the negative control. Non-motile nematodes were counted under the microscope after 24 h. To test for nematicidal effects, the incubated suspension was diluted 1:10 in M9 buffer and seeded on NGM agar plates containing *E. coli* OP50. The Petri dishes were checked for vital nematodes after 4 days of incubation at room temperature.

## Results

### Characterization of the *N. vespilloides* Gut Microbiome

**FIGURE 1:**
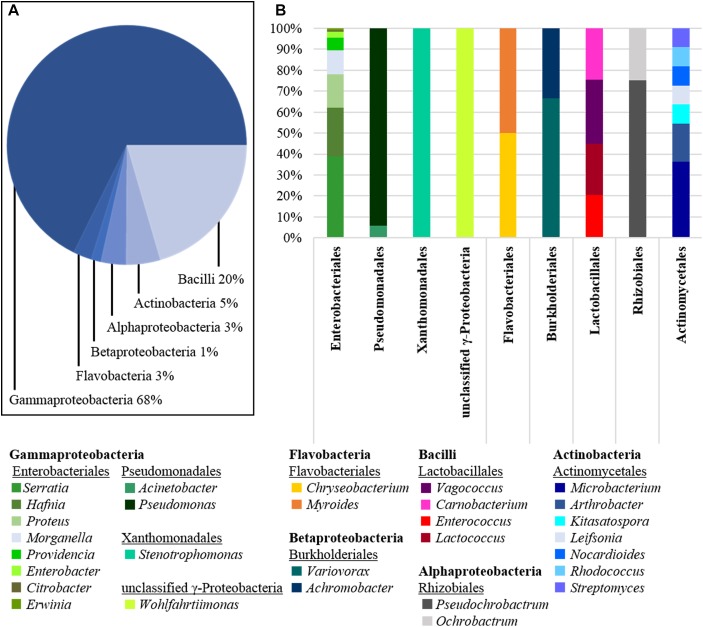
Diversity of cultivated bacteria isolated from dissected beetle guts. **(A)** The class of Gammaproteobacteria is the most abundant one isolated with 68% of all cultures obtained, followed by Bacilli (20%) and Actinobacteria (5%). Further, also Flavobacteria (3%), Alphaproteo- (3%), and Betaproteobacteria (1%) were cultivated. **(B)** The class of Gammaproteobacteria is represented by the orders of Enterobacteriales, Pseudomonadales, Xanthomonadales and a single unclassified genus (*Wohlfartiimonas*). Enterobacteriales was the order with the highest species diversity isolated. The second prominent phylum of Bacilli was represented by only four genera of the order Lactobacillales. The phylum Actinobacteria was represented by the order of Actinomycetales. Compared to the number of isolates, the species diversity within this order was surprisingly high, although other more abundant phyla appeared to be much less diverse.

After the dissection process and microbial cultivation, we were able to pick more than 400 isolates from the gut of *N. vespilloides*. Nevertheless, no distinct differences between microbial cultivates from wild caught or bred beetles were observed. The obtained isolates were checked for contaminations and identified by colony PCR and Sanger sequencing (Eurofins, Germany). A sufficient read length of the 16*S* rDNA gene was ensured by the assembly of the forward and reverse reads to a total of ∼ 1300 bp. The results were analyzed by BLASTn (NCBI), de-replicated and sorted according to the genus. The cultivated diversity is depicted in Figure 1A. We generated bacterial isolates from the Proteobacteria (∼ 3% Alphaproteobacteria, ∼ 1% Betaproteobacteria and ∼ 68% Gammaproteobacteria), ∼ 3% Flavobacteria, ∼ 20% Bacilli and ∼ 5% Actinobacteria. Enterobacteriales was the most abundant order, to which ∼ 49% of all bacterial isolates belonged. Within the order Enterobacteriales, *Serratia* (∼ 39%), *Hafnia* (∼ 23%), *Proteus* (∼ 16%) and *Morganella* (∼ 11%) were the most prevalent genera (Figure 1B). Furthermore, the genera *Pseudomonas* (order: Pseudomonadales) and *Stenotrophomonas* (order: Xanthomonadales) were also strongly represented in the phylum Gammaproteobacteria. Other prevalent genera included *Vagococcus* and *Carnobacterium* as well as *Enterococcus* and *Lactococcus* (order: Lactobacillales). The genera listed above accounted for more than 70% of all cultivated isolates. In addition to these major genera, we also cultivated *Enterobacter*, *Citrobacter*, and *Erwinia* (order: Enterobacteriales) and *Acinetobacter* (order: Pseudomonadales). The order Flavobacteriales was solely represented by the genus *Chryseobacterium*, although the latter was less prevalent than the other genera. We were also able to cultivate *Variovorax* and *Myroides* as representatives of the order Burkholderiales, and *Wohlfartiimonas*, which has not yet been assigned to an order.

### Antimicrobial Activity of the Chemical Extracts

**FIGURE 2:**
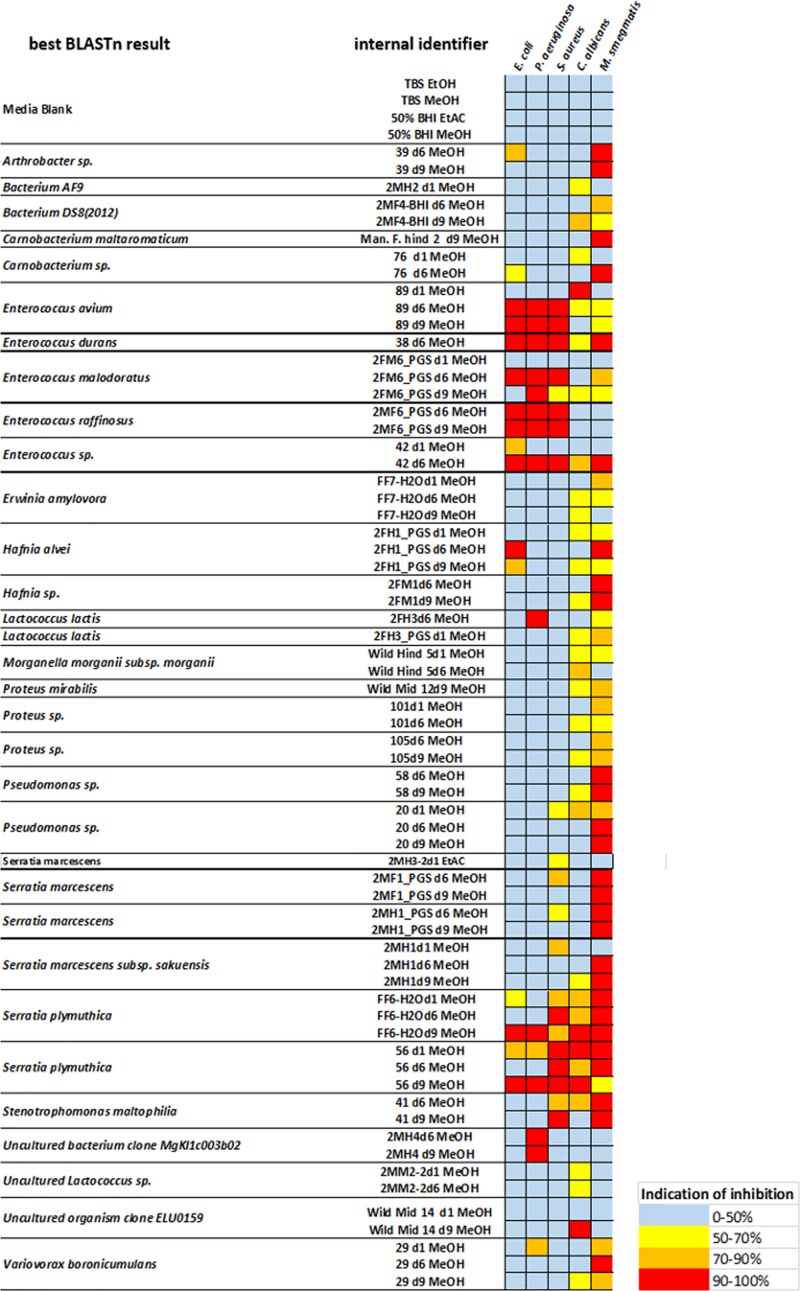
Antimicrobial activity of selected *N. vespilloides* gut bacteria against five representative bacterial pathogens. The color key indicates the percentage of growth inhibition of *Escherichia coli* ATCC 25922, *Pseudomonas aeruginosa* ATCC 27853, *Staphylococcus aureus* ATCC 25923, and *Candida albicans*. Single inhibition effects of *Mycobacterium smegmatis* are not displayed here because of the high amount of positive results. Column 1 displays the best BLAST result of the 16S analysis, column 2 contains the internal strain names, including the duration of fermentation (e.g., d6 = 6 days of fermentation) and the solvent used for extraction. On the right side, a heat map displays the range of antimicrobial effects observed.

Fermentations were established with several representative species. Cell suspensions were harvested on days 1, 6, and 9 for chemical extraction and analysis to ensure we did not miss the late induction of genes responsible for potential bioactive compounds under nutritional stress, such as carbon limitation. After chemical extraction and concentration, the crude extracts were screened against selected human-pathogenic Gram-positive and Gram-negative bacterial strains, as well as a clinically relevant strain of *Candida albicans* to identify antimicrobial activity. More than 800 crude extracts were screened for growth inhibition of test strains, revealing a broad spectrum of bioactivities. Almost none of our ethyl acetate extracts showed inhibition, so the effects of our methanol extracts were prioritized and a selection is summarized in Figure 2. A large set of families within the orders of *Micrococcales*, *Enterobacteriales*, and *Lactobacillales* with different levels of antimicrobial activity against our screening strains were observed. The broadest antimicrobial activity was found in the extracts of strains 89, 38, 42 belonging to the genus *Enterococcus* and the strains FF6-H_2_O and 56 belonging to the genus *Serratia*. Crude extracts of these two strains displayed strong inhibition of all strains tested. The extracts of strains 89, 38, 42 showed potent antimicrobial effects against *E. coli* as well as *P. aeruginosa* and *Staphylococcus aureus*, with more than 90% growth inhibition and at least 60% inhibition of *C. albicans*. The crude extracts of the genera *Pseudomonas* showed the highest bioactivity. Almost 20% of the observed bioactivities were derived from *Pseudomonas*, followed by *Enterobacter* and *Serratia*, both accounting for ∼ 12% of the total activity. Overall, 71% of all methanol crude extracts were capable of inhibiting of *M. smegmatis*. In contrast, selective inhibition of *C. albicans* has only been rarely observed. One of those examples is the isolate Wild Mid 14 (Figure 2), obtained from the midgut of a wild living beetle. Only 11% of all extracts displayed antimicrobial activity against Gram-negative bacteria. Thereof, the genera *Enterococcus* and *Serratia* yielded extracts that exhibited pronounced anti-Gram-negative activity.

**FIGURE 3:**
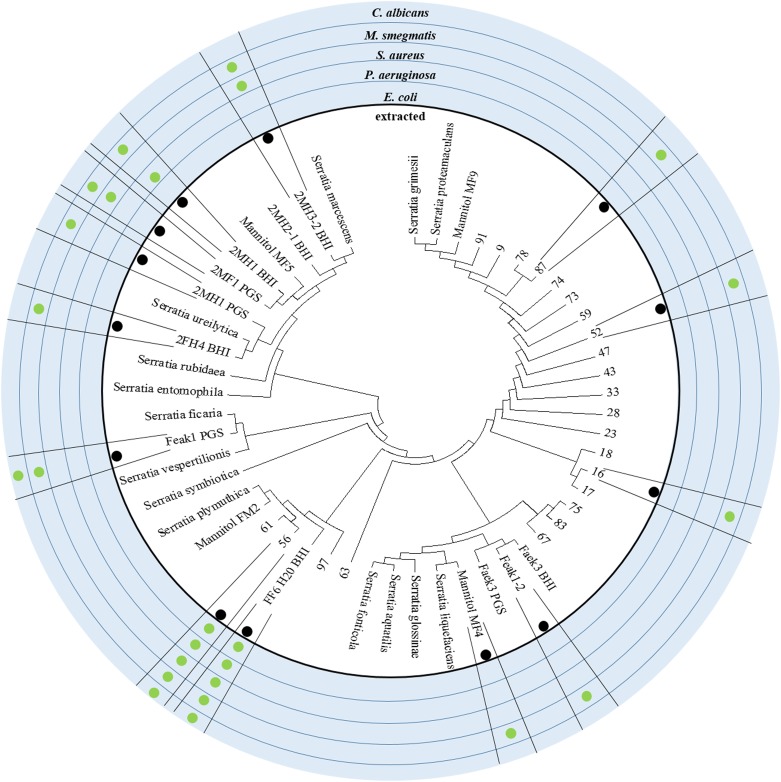
Phylogeny of the *Serratia* isolates and their antibiotic active crude extracts. The alignment of 16S rDNA sequences with *Serratia* reference strains from the NCBI database is shown here as a maximum likelihood phylogenetic tree. The outer circles indicate if the isolated strain was tested for antimicrobial effect and against which pathogen an effect could be reported. Strains such as FF6-H_2_0 and 56 display outstanding, broad spectrum antimicrobial activity and group around the reference strain *S. plymuthica*.

As mentioned above, our microbiome cultivation included a relatively large number of different *Serratia* species, some of which caused the complete inhibition of our screening strains. The broadest inhibitory effects were observed with the crude methanol extract obtained after 9 days of cultivation of the strains FF6-H_2_O and 56, both identified as *Serratia plymuthica*. Several more antimicrobial effects could be observed with the crude extracts derived from additional *Serratia* isolates. The most frequently inhibited pathogen was *M. smegmatis*, because all tested *Serratia*-derived extracts displayed bioactivity. However, inhibitory effects against *C. albicans* were rare. To correlate bioactivity and phylogeny of the *Serratia* species obtained, the observed growth inhibitions were plotted to a maximum-likelihood phylogenetic tree (Figure 3). Partial 16*S* sequences as well as reference 16*S* sequences of *S. grimesii*, *S. proteamaculans*, *S ficaria*, *S. vespertiliones*, *S. entomophilia*, *S. symbiotica*, *S. plymuthica*, *S. liquefaciens*, *S. glossinae*, *S. fonticola*, *S. aquatilis*, *S. rubidaea*, *S. ureilytica* and *S. marcescens* (all NCBI type strains) were used to generate this phylogenetic tree. The strains FF6-H_2_O and 56, with their broad antimicrobial activity, were identified as *S. plymuthica*. Both strains belong to the clade of the reference *S. plymuthica* strain and we are currently working on the identification of the active compounds in these extracts. Several further isolates showed diverse antimicrobial effects. For example, the isolate Faek1 derived from the anal secretions of *N. vespilloides* displayed antifungal activity against *C. albicans*.

**FIGURE 4:**
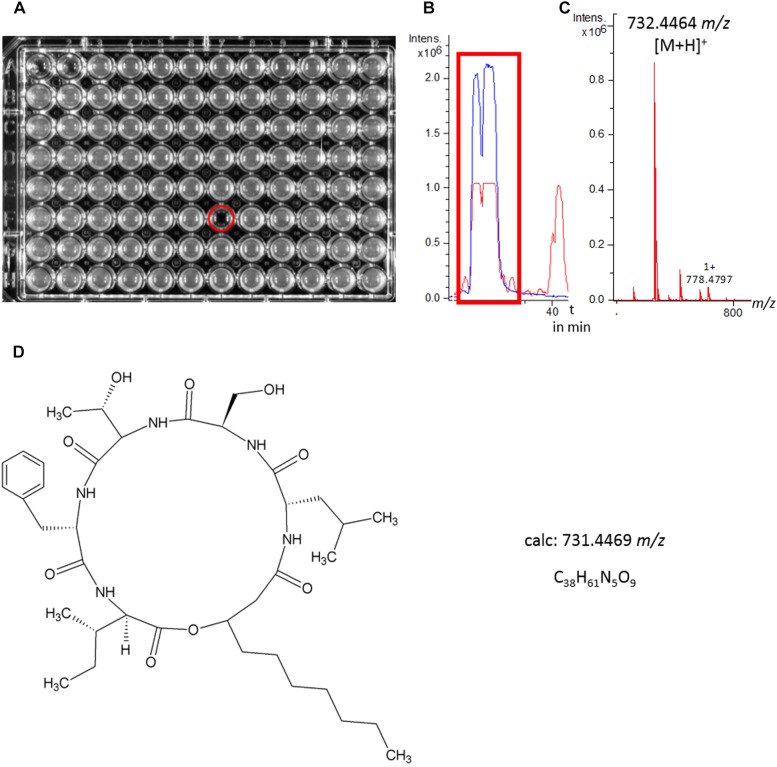
Identification of serrawettin W2. **(A)** HPLC fraction collection and biological tests for antimicrobial activity against *Staphylococcus aureus* resulted in the identification of a single active fraction. **(B,C)** LC-MS analysis revealed a major peak in the base peak chromatogram (red line) of the crude extract as the active fraction, *m/z* = 732.4464 [M+H]^+^. The extracted ion chromatogram (blue line) *m/z* 732.43–732.46 highlights the peak segment with antimicrobial activity. **(D)** Structure of serrawettin W2 (C_38_H_61_N_5_O_9_), a cyclodepsipeptide with known antimicrobial activity and a *m/z* of 731.4469 [M]^+^.

Isolate 2MH3-2 (*S. marcescens*) was chosen for further investigation due to its remarkably strong inhibition of *Staphylococcus aureus* ATCC 25923 (MSSA). To determine the structure of the antimicrobial component, the bioactive crude extract was fractionated by HPLC for bioactivity testing, revealing a single fraction that completely inhibited this pathogen (Figure 4A). Analysis of this fraction revealed one dominant peak in the base peak chromatogram (Figure 4B), which corresponded to *m/z* 732.4464 [(M+H)^+^] as determined by QqTOF-ESI-HRMS (Figure 4C). Large-scale fermentation (5 l) was therefore carried out to isolate the active compound. Initial fractionation by flash chromatography and subsequent bioactivity-guided purification of the inhibitory fraction by HPLC led to the isolation of the target molecule. NMR spectroscopy, focusing on the ^1^H and HSQC spectra, revealed five α-H signals at *δ*_H_, namely 4.61 (Phe-αH), 4.52 (Leu-αH), 4.37 (Ser-αH), 4.18 (Ile-αH) and 4.11 (Thr-αH), thus indicating the presence of a pentapeptide. This result was confirmed by HMBC spectroscopy. The aliphatic protons resonated between *δ*_H_ 2.61 and 1.16, suggesting the presence of a lipopeptide. The prominent pseudomolecular ion *m/z* 732.4464 ([M+H]^+^) in the QqTOF-ESI-HRMS spectrum agreed with the calculated *m/z* of 732.4469 (Δ *m/z* 0.0005). The molecular formula C_38_H_61_N_5_O_9_ was therefore assigned. Combining the QqTOF-ESI-HRMS and NMR data, the compound was unambiguously identified as serrawettin W2, a cyclic pentadepsipeptide (Figure 4D) that has previously been isolated from other strains of *Serratia marcescens* (Matsuyama et al., 1992). The purified compound showed no antibiotic activity against *E. coli* ATCC 25922 or the *E. coli* Δ*tolC* mutant. Notably, the MICs against the four Gram-positive test strains were in each case 4 μg/ml (Table 1).

**Table 1:** Minimal inhibitory concentrations (MICs) of pure serrawettin W2 against representative Gram-positive and Gram-negative bacteria.

Test strain	Accession number	Genotype	MIC (μg/ml)
*E. coli*	ATCC 25922	wild type	>128
*E. coli*	ATCC 25922	Δ*tolC* mutant	>128
*B. subtilis*	DSM 10	wild type	4
*S. aureus*	ATCC 25923	MSSA	4
*S. aureus*	ATCC 33592	MRSA	4
*L. monocytogenes*	DSM 20600	wild type	4


**FIGURE 5:**
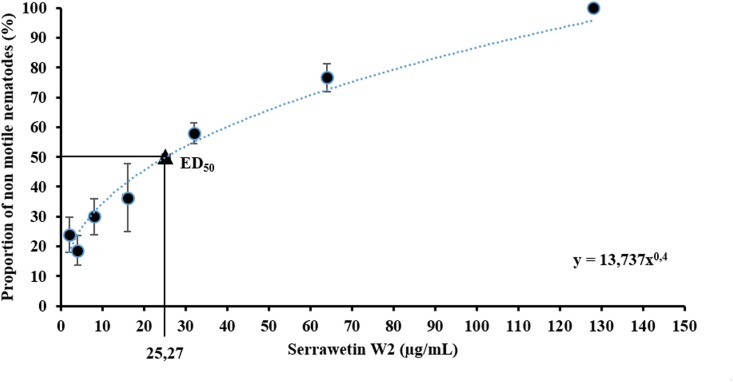
Nematode motility assay with serrawetin W2. Non-motile nematodes were counted under the microscope, and the abundance of non-motile nematodes was calculated. By displaying the experimental data (average and standard error), a resulting effective dose (ED_50_) was extrapolated.

Serrawettin W2 was previously identified as a nematode repellent (Pradel et al., 2007) but we hypothesized that it may also act as a nematicide, based on the need to suppress competing and parasitic nematodes transmitted to carcasses attended by *N. vespilloides*. We therefore conducted live-dead assays by exposing the model nematode *C. elegans* to a broad range of serrawettin W2 concentrations in technical triplicates. We observed 100% immotility at a concentration of 128 μg/ml, allowing us to extrapolate an ED_50_ value of 25.27 μg/ml based on an exponential trend line (Figure 5). Our results showed that serrawettin W2 is nematostatic at 128 μg/ml, but there were also fewer survivors compared to the control treatment, indicating the potential for nematicidal activity. Accordingly, when we seeded nematodes on NGM agar covered with a lawn of *E. coli* and exposed them to 256 μg/ml serrawettin W2, we observed a complete absence of feeding tracks (indicating live worms), confirming that the antibiotic is nematicidal at this higher concentration. We therefore consider serrawettin W2 primarily as a nematostatic compound but as a nematicide at concentrations greater than 128 μg/ml.

## Discussion

The larval and adult guts of the burying beetle *N. vespilloides* harbor a diverse microbial community including the Firmicutes, Proteobacteria and ascomycetous yeasts of the genus *Yarrowia*. Certain members of the microbiome are transmitted to the carcass via anal secretions (Vogel et al., 2017). This seeding strategy was recently proposed to facilitate the suppression of microbial competitors based on evidence of metabolic cooperation between the host and its microbiome for digestion, detoxification and defense (Duarte et al., 2018). Shukla et al. (2018b) recently corroborated this hypothesis and underlined the pivotal role of symbionts representing the genus *Serratia* in the preservation of carcasses prepared by burying beetles.

Using a distinct experimental approach by cultivating and characterizing bacterial strains isolated from the gut of *N. vespilloides*, we confirmed in this study the composition of its gut microbiota. Inspired by the hypothesis that the core microbiome of *N. vespilloides* contains bacteria, which defend the host against environmental microbes associated with the carcass and taken up with the diet, we screened the supernatants of gut bacteria cultures against a set of human pathogens to identify antimicrobial compounds. Subsequently, we observed a large variety of different antimicrobial effects, partly summarized in Figure 1. In this context, we observed strong activity against *S. aureus* in supernatants from an isolate identified as *S. marcescens*. Subsequent characterization of the antimicrobial compound resulted in its identification as serrawettin W2. The latter molecule consists of five amino acids and an additional fatty acid residue (Matsuyama et al., 1992). This cyclic lipodepsipentapeptide displayed potent activity against the Gram-positive bacterium *S. aureus* ATCC 33592 (MRSA) in our growth inhibition assays. Shukla et al. (2018a) revealed the presence of staphylococci on untended carcasses whereas carcasses prepared by burying beetles are devoid of these bacteria. This agrees with our finding that serrawettin W2 has potent inhibitory activity against *Staphylococcus aureus*. The most prominent group of *Serratia* antibiotics are undoubtedly the red pyrrole alkaloid prodigiosin and its derivatives (Williams, 1973; Fürstner, 2003; Lapenda et al., 2015). Despite this, none of the *Serratia* isolates investigated in this study formed a red pigment. Apart from these pigments, carbapenem antibiotics have been reported that are produced by *Serratia* species (Bycroft et al., 1987). A *Serratia* sp. was reported as the producer of six cyclodepsipeptides with distinctive anti-mycobacterial activity. Notably, serratomolides A–F displayed antibacterial activity against *Mycobacterium diernhoferi* and additional rapidly growing mycobacteria, but no inhibition of other human pathogenic Gram-positive and Gram-negative bacteria was observed (Dwivedi et al., 2008).

Furthermore, pure serrawettin W2 showed strong nematostatic effects against the model organism *C. elegans*. This indicates that serrawettin W2 is not only a nematode repellant as previously reported (Pradel et al., 2007) but also a potent nematostatic molecule. The ability to deter nematodes is of particular importance to burying beetles, which are associated with parasitic nematodes, some of which are exclusively associated with *N. vespilloides* (Richter, 1993). The nematode dauer juveniles use the beetles’ gut as a vehicle to transfer them to carrion, where they feed and reproduce and thus achieve *trans*-generational transmission from parent to larvae through the carcass surface and direct parental interactions. The dauer juveniles of the offspring generation migrate to the genitalia and the gut of the hatching beetles to use them for transfer to the next carcass (Richter, 1993). Transcriptomic analysis of the feeding cavity of beetle-prepared carcasses (where larvae reside and feed) indicates that the nematodes are not only prevalent along with *N. vespilloides*, but are also metabolically active, competing for carcass nutrients with the beetle larvae (Vogel et al., 2017). Recently, another phoretic nematode associated with *N. vespilloides* (*Rhabditoides regina*) was described as a parasite because it negatively affected the beetle’s brood size, larval survival and larval mass (Wang and Rozen, 2019). Because *Rhabditoides* nematodes can influence carcass health, larval survival and beetle fitness, we postulate that microbes in the *N. vespilloides* gut producing serrawettin W2 may help the beetles to suppress nematode populations in the gut as well as on the carcass surface.

The discovery of antibiotic-producing bacteria in the gut of *N. vespilloides* indicates the presence of microbial symbionts contributing to the control of bacteria and nematodes, which could harm the eggs laid near by the carcass (Jacobs et al., 2014) and compete with the larval offspring to exploit the carcass as a highly nutritious but ephemeral food source. The results presented here fit well with the proposed strategies for managing rival bacterial communities in *N. vespilloides* (Duarte et al., 2018).

Our study suggests that the strategy of outsourcing defense against pathogens and parasites to bacterial symbionts is more widespread in beetles than previously thought (Dose et al., 2018; Flórez et al., 2018; Schmidtberg et al., 2019). The symbiotic microbiome of beetles therefore offers an untapped resource for the discovery of novel natural products with activity against human pathogens and parasites.

## Data Availability

Sequences of *Serratia* isolates are deposited in GenBank (MK386907-MK386931 and MK391500-MK391513).

## Author Contributions

HV, TS, and AV designed the experiments. PH performed the microbial and molecular work, processed the samples, analyzed the data, and prepared the manuscript. YL isolated the compound and performed the structure elucidation. TD conducted the LC-MS experiments, analyzed the data, and prepared the manuscript.

## Conflict of Interest Statement

The authors declare that the research was conducted in the absence of any commercial or financial relationships that could be construed as a potential conflict of interest.
